# Supplementary figures and images for: The COVID-19 Pandemic Is Associated with Reduced Survival after Pancreatic Ductal Adenocarcinoma Diagnosis: A Single-Centre Retrospective Analysis
Source: J Clin Med. 2022 May 4;11(9):2574. doi: 10.3390/jcm11092574 (PMC9105306; doi:10.3390/jcm11092574)

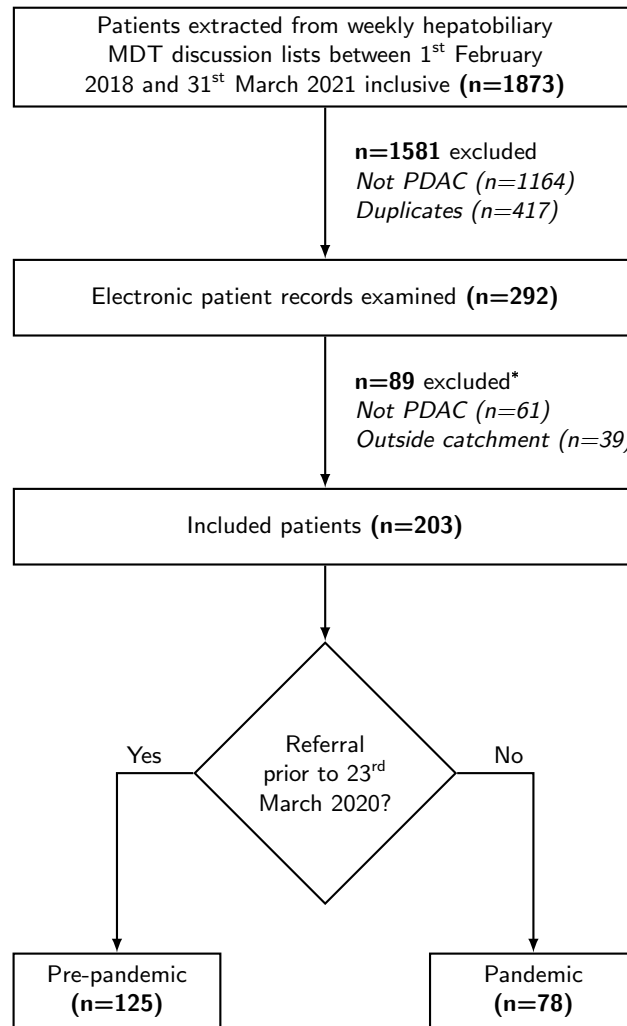

\* 11 excluded on both criteria

Figure S1: STROBE flow-chart of study case selection

Supplement: Supplementary file 1 [file jcm-11-02574-s001.zip › jcm-1682322-suppl proof done.pdf]
